# King Edward VII

**Published:** 1910-05-14

**Authors:** 


The Hospital

A Journal of

The Medical Sciences and Hospital Administration.

New Series. No. 169, Vol. VII. [No. 1241, Vol. XLVIII.] SATURDAY, MAY 14, 1910.

### KING EDWARD VII.

The nation mourns for a great and noble monarch. High and low, rich and poor, all creeds, all parties, for once are united in one common sentiment at this time of our loss. Alike they voice the feelings of personal sorrow and grief, for our late Sovereign had inspired the regard of his subjects no less by his kingly dignity than by his tact, his sympathy, and the graciousness with which all classes of men and women were made equally to feel themselves his subjects and the objects of his royal solicitude. The loss which we have sustained is also felt to the full by our loyal fellow-subjects under the Imperial Crown, the inhabitants of our great colonies and dependencies across the seas. Even in foreign countries the expressions of regret and sympathy which have been freely evoked contain a note of real and genuine concern; for it is recognised abroad almost as fully as at home that as long as Edward the Peacemaker lived the awful catastrophe of a European war was hardly possible.

Amid the tributes of affectionate regard and respect for our late King which have flowed in from all sides, both in Great Britain, the Empire, and the world at large, it is just and seemly that the medical profession should be especially represented. Not only as ordinary citizens do we mourn the death of a King who was always first and foremost the guardian of his people's happiness and well-being. Even more than other sections of the community have we reason to know and appreciate the kind heart, the wise head, and the practical character of a King who helped the hospitals—and therefore the poor and needy in their distress, the halt, the maimed, and the afflicted—as no king has ever helped them before. Amid all the titles bequeathed to this puissant prince by his ancestors and the orders of chivalry conferred upon him at home and abroad, there were none better deserved and more thoroughly lived up to than that of Sovereign Head and Patron of the Order of St. John of Jerusalem in Britain, and few which gave him such pleasure and satisfaction to possess.

For the peculiar favour which King Edward always displayed towards the medical profession no doubt a combination of circumstances has been responsible. First of all, the humanity which impelled him always to be mindful of the humblest of his people in their hours of sickness and misery. Hence the constant care with which he fostered hospitals and charities of all kinds designed to aid the sick and suffering who could not of their own resources command the attention requisite for a restoration to health. In thus occupying himself, the King was of necessity brought intimately into contact with many members of the medical profession; oftenest, of course, with consulting surgeons and physicians of high standing and mature reputation, but also in some measure with a good many of what may be called without offence the rank and file of the profession. Thus our late Sovereign was acquainted with medical men perhaps to a greater degree than with the members of other professions or callings among his subjects. Then, again, King Edward was himself no stranger to illness and accident. Well indeed had he cause to know the infirmities and ills that flesh is heir to, for they spare not kings any more than humble folk. Nearly forty years ago his late Majesty lay sick almost unto death of typhoid fever, and they whose memory carries them back to those weeks when his life trembled in the balance can never forget the solemn and chastened joy with which his people returned their grateful thanks when at last he emerged victorious from the long struggle. Later on a fractured patella confined him to bed for a long period. Already middle-aged when this unfortunate accident occurred, the results of treatment were so satisfactory that the fracture had long since passed into oblivion, so little was there in the King's gait, so little curtailment of his activities, to remind any one of a lesion which might easily, for all the skill and care of his advisers, have left some permanent disability.

But of all the King's first-hand experiences of the sick-bed, the most dramatic, as it was the latest and the most serious, was the attack of appendicitis for which he underwent an operation by Sir Frederick Treves upon the day that should have witnessed his coronation. It is an old story now, but one not to be forgotten in estimating the character of King Edward and the hold which he had over the hearts of his people. Attacked by symptoms of this painful malady a week or so before the great pageant was to be solemnised, his Majesty refused even to take the enjoined rest, but continued to drive about that his subjects might not guess any hint of impending calamity. His one thought was not to disappoint his guests and his people, to go through with the Coronation, and then to be ill, if ill he must be, afterwards. We all know the result, and how nearly he accomplished the plucky, if unfortunate, resolution he had made. On the very day fixed for the ceremony his condition became so much worse that the inevitable could no longer be delayed, and the surgeon's knife was demanded to relieve the condition from which no race or no class is exempt.

It may have been the memory of the many weary hours thus spent in sickness and convalescence that quickened in some degree the late King's interest in hospitals, their finances, progress, and management. But indeed his kind heart needed no such personal incentive to consider the needs of his countrymen. His sympathies were so large and so wide that every hospital which was doing good work had in him a friend and a patron. The King Edward VII. Hospital Fund everyone has heard of; but not everyone understands that, but for its institution and the energy with which the King promoted its interests, probably several of the London voluntary hospitals would have had to close their doors. Certainly no account of the late reign will be complete without an important chapter devoted to the care with which he fostered the Hospital Sunday and Saturday Funds, the Edward VII. Sanatorium, the Edward VII. Hospital for Officers, and the numberless other charitable institutions for the relief of the sick poor on behalf of which his influence was always exercised at need. Fortunate indeed is it for the future of the hospitals that his son and heir has taken as vivid and practical an interest in them as did his father, whose death the nation, and the medical profession especially, now so sincerely mourns.

